# The nasal cavity and its olfactory sensory territories

**DOI:** 10.3389/fnana.2015.00031

**Published:** 2015-03-18

**Authors:** Ignacio Salazar

**Affiliations:** Unit of Anatomy and Embryology, Department of Anatomy and Animal Production, Faculty of Veterinary, University of Santiago de CompostelaSantiago de Compostela, Spain

**Keywords:** main olfactory epithelium, vomeronasal epithelium, ganglion of Grüneberg

The sense of smell is more complex than previously believed. For different reasons, the receptors traditionally thought of having the ability to identify chemical olfactory signals were exclusively confined to the epithelium of the mucosa lining both the posterior part of the walls of the nasal cavity and the ethmoturbinates. However, at the end of the nineteenth century, the vomeronasal organ (VNO) was demonstrated to have its own sensory epithelium, able to perform a similar function (Retzius, 1894). In later years, a small and isolated area of olfactory epithelium was detected in the nasal septum (Rodolfo-Masera, 1943); this structure was denominated septal organ. Later evidence showed than a group of cells located on the superior and anterior part of the nasal vestibuli (Grüneberg, 1973) also had a functional role within the olfactory system (Fuss et al., 2005). These findings led to being currently accepted that in certain macrosmatic animals the olfactory sensory receptors are located in four different regions of the nasal cavity: the main olfactory epithelium (MOE), the vomeronasal epithelium (VnE), the septal organ (SO), and the ganglion of Grüneberg (GG). Besides, a subdivision of both the MOE and the VnE can be done into four and two areas respectively. Following a morphological criterion four/eight structures integrate the olfactory subsystems (OSbS) (Breer et al., 2006). However, the axonal projections of those structures onto the olfactory bulb have been observed to show overlapping (Munger et al., 2009), and the concept of OSbS should be limited to the morphological location of the mentioned structures. That point of view has been used in this Research topic.

To begin to understand the sense of smell as a whole in the mammalian class, one of the biggest challenges we are confronted with is to find answers to some basic and critical questions as, for instance: what are exactly the GG, the VNO and the SO, and what are their precise functions? How are the connections between them and how does each of them interact with the MOE? What happens if one or more than one of such structures is absent in a particular species? From an evolutionary perspective, it is reasonable to assume that partial or total response to these questions and others can be found, but it would require having the genome of the species, and a deep knowledge of the morphological characteristics of each structure.

Many recent studies have contributed to the progress in the knowledge of the sense of smell, and one of the most relevant is the work of Buck and Axel (1991), where the authors laid the molecular foundations for odor recognition (see also Axel, 2005; Buck, 2005). Another recent and significant work concerns the implication of the main olfactory subsystem (MOS) in the recognition of pheromonal signals, performed by three different laboratories and published in the same year (Boehm et al., 2005; Mandiyan et al., 2005; Yoon et al., 2005; see also Baum and Larriva-Sahd, 2014). These papers not only demonstrated the important role MOS plays in sexual and aggressive behavior, but also revealed an anatomical projection from the MOE to LHRH neurons in the neuroendocrine hypothalamus, which regulate gonadal hormone release (Keverne, 2005). Such findings have revolutionized our understanding over how the sense of smell controls the neuroendocrine brain. The classical belief that common odors are perceived through the olfactory pathway and pheromones by the VNO is dead. Each pathway must be assessed for a putative pheromone on its own evidence (Shepherd, 2006).

This Research Topic includes, as an introduction, an interesting opinion article (Mori et al., 2014) where the authors establish a new and appealing relationship between the olfactory perception and the respiratory rhythm, being involved different regions of the central olfactory system like the olfactory bulb and some areas of the olfactory cortex. The morphology of the OSbS as a whole has been addressed by Salazar's group in two articles. The first of them presents an on-line atlas of the murine nasal cavity, freely available at http://www.usc.es/anatembriol/ (Barrios et al., 2014a); in the second, one of the most relevant results points out that the dog lacks two of the olfactory areas of the mouse, the SO and the GG (Barrios et al., 2014b).

The SO, when present, is a small patch of olfactory sensory epithelium forming an island in the respiratory epithelium, near the inferior part of the nasal septum. This organization shows resemblance with the typical distribution of both epithelia in humans (Escada et al., 2009), and is therefore the structure of the OSbS to which less attention is paid, in exact opposition to what occurs with the GG. In a nice paper Brechbühl et al. (2014), using different procedures, show the presence of the GG in the rat, hamster and gerbil, and found that those species had morphological and physiological differences.

Finally, five articles are devoted to various aspects of the VNO. Ackels et al. (2014) study the characterization of the recently discovered formyl peptide receptor family and suggest the use of the transgenic mouse strain model they employed to carry out future research about the subject. A mini review of the basal vomeronasal neuroepithelium is the contribution of Pérez-Gómez et al. (2014). In this work, the authors highlight the critical role of this part of the epithelium in different ranges of instinctive behaviors. Also in mice, as the previous reference, Cavaliere et al. (2014) establish a direct relationship between the orbital glands and the vomeronasal system by means of the detection of the exocrine gland-secreting peptides. The last two contributions deal with the genetic and evolutionary approach. In an elegant paper Hohenbrink et al. (2014) present the expression patterns of vomeronasal receptors of a species of primate, and at the same time demonstrate that a substantial number of such receptors were expressed in the MOE. Therefore, the chemosensory system in primates indicates greater functional overlap between the VNO and MOE, a very interesting finding. The contribution of Yoder and Larsen (2014) is mainly focused on the molecular-evolutionary dynamics of vomeronasal receptor genes1 in primates; the authors review and update this interesting issue, closely related to the goal of the present Research Topic.

## Conflict of interest statement

The authors declare that the research was conducted in the absence of any commercial or financial relationships that could be construed as a potential conflict of interest.
